# Portal Vein Embolization: Impact of Chemotherapy and Genetic Mutations

**DOI:** 10.3390/jcm6030026

**Published:** 2017-03-01

**Authors:** Amy R. Deipolyi, Yu Shrike Zhang, Ali Khademhosseini, Sailendra Naidu, Mitesh Borad, Burcu Sahin, Amit K. Mathur, Rahmi Oklu

**Affiliations:** 1 Interventional Radiology Service, Memorial Sloan Kettering Cancer Center, New York, NY 10065, USA; deipolya@mskcc.org; 2 Biomaterials Innovation Research Center, Division of Biomedical Engineering, Department of Medicine, Brigham and Women’s Hospital, Harvard Medical School, Boston, MA 02115, USA; yszhang@mit.edu (Y.S.Z.); alik@bwh.harvard.edu (A.K.); 3 Harvard-MIT Division of Health Sciences and Technology, Cambridge, MA 02139, USA; 4 Wyss Institute for Biologically Inspired Engineering, Harvard University, Boston, MA 02115, USA; 5 Division of Vascular and Interventional Radiology, Mayo Clinic, Phoenix, AZ 85054, USA; naidu.sailen@mayo.edu; 6 Division of Oncology, Mayo Clinic, Phoenix, AZ 85054, USA; borad.mitesh@mayo.edu; 7 Department of Radiology, Ankara Oncology Training and Research Hospital, Ankara, Turkey; bsavrans@gmail.com; 8 Division of Transplant Surgery, Mayo Clinic, Phoenix, AZ 85054, USA; mathur.amit@mayo.edu

**Keywords:** portal vein embolization, angiography, embolization, chemotherapy, mutation

## Abstract

We characterized the effect of systemic therapy given after portal vein embolization (PVE) and before hepatectomy on hepatic tumor and functional liver remnant (FLR) volumes. All 76 patients who underwent right PVE from 2002–2016 were retrospectively studied. Etiologies included colorectal cancer (*n* = 44), hepatocellular carcinoma (*n* = 17), cholangiocarcinoma (*n* = 10), and other metastases (*n* = 5). Imaging before and after PVE was assessed. Chart review revealed systemic therapy administration, SNaPshot genetic profiling, and comorbidities. Nine patients received systemic therapy; 67 did not. Tumor volume increased 28% in patients who did not receive and decreased −24% in patients who did receive systemic therapy (*p* = 0.026), with no difference in FLR growth (28% vs. 34%; *p* = 0.645). Among 30 patients with genetic profiling, 15 were wild type and 15 had mutations. Mutations were an independent predictor of tumor growth (*p* = 0.049), but did not impact FLR growth (32% vs. 28%; *p* = 0.93). Neither cirrhosis, hepatic steatosis, nor diabetes impacted changes in tumor or FLR volume (*p* > 0.20). Systemic therapy administered after PVE before hepatic lobectomy had no effect on FLR growth; however, it was associated with decreasing tumor volumes. Continuing systemic therapy until hepatectomy may be warranted, particularly in patients with genetic mutations.

## 1. Introduction

Portal vein embolization (PVE) is performed before hepatic lobectomy for primary and secondary liver malignancy to increase the size of the functional liver remnant (FLR) to avoid post-hepatectomy failure [1,2]. During the interval between PVE and surgery, tumor growth may occur, and can be mediated by several pathways. For instance, the RAS proteins, which are GTPases involved in cell signaling, are among the most common oncogenes, with KRAS mutations determining the response to certain systemic therapies [3]. PI3Ks, intracellular signal transducer enzymes, stimulate RAS pathways while inhibiting tumor suppressor pathways including the tumor protein p53 [4,5]. PVE may induce tumor growth by a variety of mechanisms, such as altered arterial supply to the liver or stimulating growth factor and cytokine pathways [6,7].

Because of the potential of tumor growth after PVE while patients await surgery, some propose administering systemic therapy during this interval [8]. However, systemic therapy may prevent hyperplasia and the growth of the FLR, reducing PVE efficacy. PVE is expected to increase the FLR volume by 25%–50%, with non-cirrhotic livers demonstrating a larger increase in volume compared with cirrhotic livers [9]. Several studies have not shown a significant impact on FLR hypertrophy by the administration of chemotherapy [9,10]. However, one study reported that FLR growth was reduced by a third by pre-surgical chemotherapy in the setting of colorectal metastasis [11], and other studies have shown a trend for reduced FLR hypertrophy in patients on chemotherapy after PVE [12,13].

The purpose of this study was to characterize the effect of PVE and cancer-related genetic mutations on tumor and liver volume changes in patients who were or were not treated with systemic therapy during the interval prior to surgical lobectomy.

## 2. Materials and Methods

In this retrospective, single institution, Health Insurance Portability and Accountability Act-compliant, Institutional Review Board-approved study, the requirement for informed consent was waived. All consecutive patients who underwent right PVE from January 2002 to December 2014 were identified using the radiology department PACS search engine.

A total of 76 patients (30F, 46M) with a mean age of 61 years (range 37–83) who underwent right PVE for right liver malignancy were included (Table 1). Patients who received chemotherapy were significantly younger (*p* < 0.001), and none had primary hepatic malignancy. Overall, etiologies included colorectal cancer (*n* = 44), hepatocellular carcinoma (HCC; *n* = 17), cholangiocarcinoma (*n* = 10), gastrointestinal stromal tumor (3), thyroid cancer (1), and lacrimal gland tumor (1).

**Figure 1:**
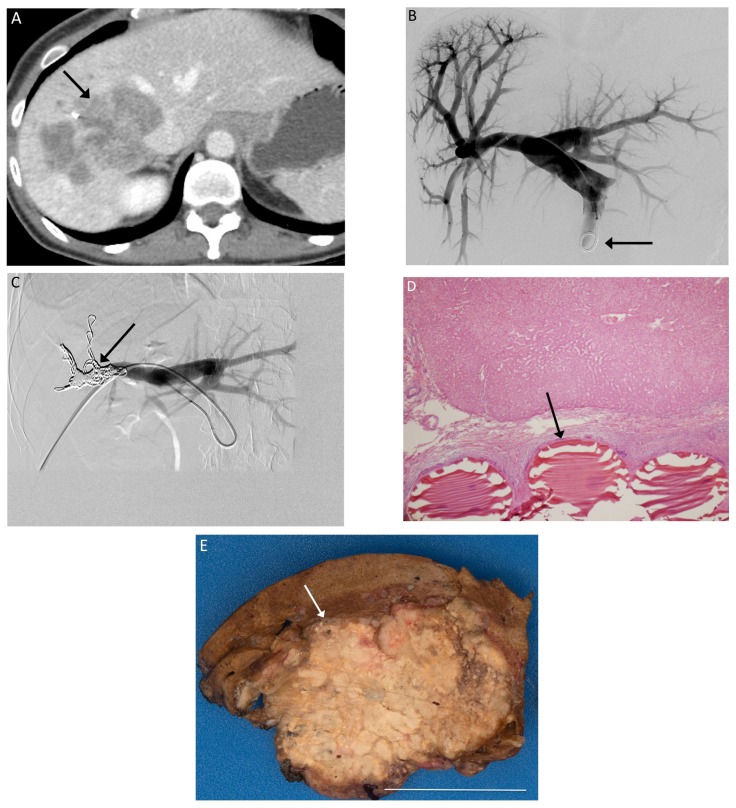
(**A**) Pre-procedure CT imaging demonstrates a right hepatic metastasis (arrow) from colorectal cancer, with a diminutive left lobe; (**B**) Tranhepatic portography (arrow) is obtained after portal access is achieved via the right portal vein; (**C**) The right portal branches have been embolized with particles and metallic coils (arrow); (**D**) 200× magnification H&E stain slide demonstrates Embosphere particles within a portal vein (arrow); (**E**) Gross specimen after right hepatectomy demonstrates particles within the embolized right hepatic lobe. White bar indicates 5 cm.

Pre-procedure magnetic resonance imaging (MRI) or computed tomograhy (CT) was performed 33 ± 5 days before PVE, whereas post-procedure MRI or CT was performed 33 ± 6 days after PVE. All PVEs involved the right portal vein prior to planned right hepatectomy. PVE was performed in standard fashion using a right lobe approach with both particles and metallic coils [14] (Figure 1). Images were assessed with TeraRecon (Foster City, CA, USA) by a radiologist blind to the patient’s treatments to calculate liver and tumor volumes before and after PVE.

Chart review revealed systemic therapy administration, genetic profiling, and comorbidities including diabetes, cirrhosis, and hepatic steatosis. Genetic profiling with SNaPshot (Thermo Fisher Scientific, Springfield Township, NJ, USA), a high-throughput PCR assay that detects over 100 gene mutations [15], was performed in 30 patients in the cohort.

Statistical analysis was performed with GraphPad Prism 6.0 (GraphPad Software, La Jolla, CA, USA), with *p* < 0.05 used for statistical significance. Data are presented as mean ± standard error. Tumor growth was calculated by subtracting the volume of the hepatic tumor(s) after PVE from the volume of the tumor before PVE, then dividing by the volume before PVE, and presented as a percentage. FLR growth was similarly calculated using the volume of the left lobe before and after PVE. Because data were not normally distributed, by Shapiro-Wilk test, Mann-Whitney U test was used to compare means. Chi-square tests were used to compare categorical data. Multiple regressions were used to assess the impact of multiple variables on tumor and FLR growth.

## 3. Results

Of the 76 patients, nine patients received systemic therapy and 67 did not (Table 2). Patients who did not receive systemic therapy had increased tumor volume (28% ± 13%) whereas patients who received systemic therapy had decreased tumor volume (−24% ± 23%) (*p* = 0.026). However, there was no significant difference in the increase in FLR between patients who received systemic therapy (28% ± 8%) compared to those who did not (34% ± 4%) (*p* = 0.645).

Because none of the patients who received systemic therapy had primary liver malignancy (cholangiocarcinoma or HCC), a subgroup analysis was performed comparing patients with metastatic liver tumors who did and did not receive systemic therapy. Patients with metastatic disease who did not receive systemic therapy had a 46% ± 20% increase in tumor volume; in contrast, patients with metastatic disease who did receive systemic therapy had a 24% ± 23% reduction in tumor volume (*p* = 0.013). In contrast, for patients with metastatic disease, there was no significant difference in FLR growth among those who did not (38% ± 5%) and did (28% ± 8%) receive systemic therapy.

Multiple regression analysis was used to assess the impact of the presence of cirrhosis, hepatic steatosis, and diabetes on changes in the FLR volume. The model was not significant (*p* = 0.538) and none of the variables were independent predictors of FLR growth (*p* > 0.20). Similarly, tumor growth was also not predicted by a model assessing cirrhosis, steatosis, and diabetes (*p* > 0.40), with no variable independently predictive (*p* > 0.10).

Of the 76 patients, 61 (80%) underwent hepatectomy as planned, on average 45 ± 3 days after PVE (Table 3). Seven patients had progression of disease precluding hepatectomy, four of which were noted intraoperatively, and three on post-PVE imaging. Of note, all seven of the patients with progression of disease precluding surgery did not receive chemotherapy after PVE before planned resection. Six patients had insufficient hypertrophy of the FLR precluding hepatectomy; four of these were noted on post-PVE imaging and two were noted intraoperatively. Of the six patients with insufficient hypertrophy, one received chemotherapy and the others did not. Of the two intraoperative cases with insufficient FLR hypertrophy, one had a partial resection as part of a new planned staged resection, and the other had a portal vein ligation. The final two patients did not undergo hepatectomy due to complications from PVE: one patient had a liver abscess because thermal ablation was also performed at the time of PVE, and the other patient had hemorrhagic shock during PVE due to an arterioportal shunt that was treated with arterial embolization. Both of these patients were too ill to undergo additional surgery.

Among the 30 patients with genetic profiling with SNaPshot, 15 were wild type and 15 had one or more mutations including *KRAS* (eight), *TP53* (five), *KIT* (one), *PI3K* (one), *MSH2* (one), and *SDHC* (one). Three patients with mutations and three patients with the wild type genotype received chemotherapy. Patients with mutations had greater increased tumor volume (77% ± 46%) compared to those without (10% ± 20%), though this difference was not significant by the Mann-Whitney U test (*p* = 0.412). Patients with mutations had similar FLR growth (32% ± 8%) compared to patients without mutations (28% ± 8%) (*p* = 0.806). Multiple regression was performed with diabetes, hepatic steatosis, administration of systemic therapy, and genetic mutation as input variables and tumor growth as the output. Though the model was not significant (*p* = 0.097), the presence of genetic mutations was the only significant independent predictor of increased tumor growth after PVE (*p* = 0.049). Among the 15 patients with mutations, 12 underwent surgical resection as planned, one did not due to progressive disease and two did not due to insufficient hypertrophy. Among the 15 wild-type patients, 11 underwent surgery as planned, two did not due to progressive disease, and two did not due to insufficient hypertrophy.

## 4. Discussion

Chemotherapy given after PVE and before hepatic lobectomy had no effect on FLR growth. However, patients who did not receive chemotherapy had significant increases in tumor volume, whereas those who continued chemotherapy had decreased tumor volume. Theoretically, chemotherapy could impact FLR growth by a number of mechanisms. Embolization stimulates hepatocyte growth via a number of mechanisms, including stimulation of proinflammatory cytokines and growth factors that are directly or indirectly suppressed by systemic immunotherapies and chemotherapies [16]. Indeed, FLR growth was reduced by a third when chemotherapy was given to patients undergoing PVE for colorectal metastasis [11]. In the present study, there was a slight decrease in FLR growth in patients on chemotherapy compared with those not on chemotherapy (28% vs. 34%); however, as in other prior studies, this trend did not reach significance [12,13].

Several prior studies have suggested that PVE could induce tumor growth by a variety of mechanisms, such as growth factor and cytokine pathway alterations or changes in the arterial supply to the liver [6,7]. Furthermore, embolization activates pre-inflammatory cytokine pathways and mediators, including interleukin-6 and tumor necrosis factor-alpha, which then could trigger growth of tumors both within the liver and at distant sites [16,17]. Thus, several papers have described the progression of disease while chemotherapy is withheld for PVE and surgery [6,7,8,10]. However, it is also possible that existing tumors are not specifically triggered to grow, but rather are merely no longer inhibited because the chemotherapy is withheld. In our study, we observed that withholding chemotherapy was associated with tumor growth, whereas administering chemotherapy was associated with tumor volume reduction.

A subset of patients underwent genetic profiling with SNaPshot analysis. The mean tumor growth was 77% among patients with genetic mutations, compared with only 6% for wild-type patients. The two most commonly detected genetic mutations in this study were *KRAS* and *TP53*. Mutations in *KRAS*, a gene encoding a component of the epidermal growth factor receptor signaling network, confer resistance to certain systemic therapies including cetuximab [18], and are associated with significantly reduced survival [19]. Mutations in *TP53*, a gene encoding a tumor suppressor protein [20], are associated with increased recurrence and decreased survival in colorectal cancer [21]. A previous study demonstrated that *KRAS* and *TP53* mutations were associated with reduced survival after radioembolization, an effect possibly mediated by angiogenic derangements including intratumoral vascular shunting [22], which could impair the delivery of therapeutic agents [23]. Because patients with genetic mutations have more rapid tumor growth and metastasis, withholding targeted systemic therapies may put such patients at greater risk for progression of disease while awaiting surgery.

The primary study limitations are its retrospective nature and the relatively low sample size. Only a fraction of patients received chemotherapy and underwent genetic profiling. There may be undetected bias that influenced which of the patients received systemic therapy or underwent SNaPshot testing. The variation in genetic mutations between patients, and in the type of chemotherapies delivered to each patient, precludes assessing the impact of specific genes and chemotherapies. Also, no patient with primary liver malignancy received chemotherapy, so it is not possible to characterize the potential effect of systemic treatment on FLR and tumor growth for these patients. The smaller sample size for patients receiving chemotherapy may have limited our ability to detect a significant difference between chemotherapy and no chemotherapy groups regarding changes in FLR. Power analysis reveals that 490 patients would be necessary to detect such a difference with 80% power. Additionally, future studies could assess measures of liver function before and after resection, and post-operative complications and survival, to assess the impact of pre-surgical systemic therapy on functional outcomes beyond the impact on hepatic and tumor volumes.

## 5. Conclusions

In summary, administration of systemic chemotherapy after PVE before hepatic lobectomy did not have a significant impact on FLR growth, but did lead to reduced tumor progression or even tumor shrinkage. Given these findings, chemotherapy appears to be safe and effective and should likely not be withheld, particularly in patients with genetic mutations that may be prone to more rapid tumor progression.

## Figures and Tables

**Table 1:** Demographics and pre-procedural characteristics of patients who did and not receive chemotherapy between portal vein embolization (PVE) and hepatic lobectomy.

Study Population	No Chemotherapy	Chemotherapy	*p* Value
Number of patients	67	9	
% Male	61%	56%	0.745
Age	63 ± 1 year	50 ± 3 year	<0.001
Pre-PVE FLR	35 ± 1%	35 ± 3%	0.960
Tumor volume	115 ± 31 cc	105 ± 50 cc	0.910
Number of tumors	3.2 ± 0.3	5.7 ± 1.4	0.122
Etiology			0.131
HCC	25%	0	
Cholangiocarcinoma	15%	0	
Colorectal cancer	54%	89%	
Other metastasis	6%	11%	

**Table 2:** Systemic therapy administered to patients after PVE before surgical lobectomy.

Age	Gender	Etiology	Chemotherapy Regimen
66	Male	Colorectal cancer	FOLFOX/bevacizumab
44	Female	Colorectal cancer	5FU
39	Female	Colorectal cancer	FOLFIRINOX
55	Female	Colorectal cancer	FOLFOX
40	Male	Colorectal cancer	FOLFOX/bevacizumab, FOLFIRI/cetuximab
50	Male	Colorectal cancer	FOLFOX
61	Female	Colorectal cancer	FOLFOX
51	Male	Colorectal cancer	FOLFIRI, bevacizumab, 5-FU
40	Male	GIST	Sunitinib

**Table 3:** Outcomes after PVE.

Volume Changes after PVE	No Chemotherapy	Chemotherapy	*p* Value
% Change in tumor volume	28% ± 13%	−24 ± 23%	0.026
% Daily change in tumor volume	0.5% ± 0.2%	−0.3% ± 0.3%	0.092
% Change in FLR volume	34% ± 4%	28% ± 8%	0.740
% Daily change in FLR volume	0.7% ± 0.1%	0.4% ± 0.1%	0.287
% Cases that underwent hepatectomy	79%	88%	0.489
